# Quantifying Disease Severity in Health Technology Assessment in Japan: A Retrospective Analysis Using Quality-Adjusted Life-Year Shortfalls

**DOI:** 10.36469/001c.147469

**Published:** 2025-12-02

**Authors:** Miwa Enami, Akira Yuasa, Shunya Ikeda

**Affiliations:** 1 Japan Access & Value, Pfizer Japan Inc., Tokyo, Japan; 2 Department of Public Health, School of Medicine International University of Health and Welfare, Narita, Japan.

**Keywords:** cost–effectiveness analysis, quality-adjusted life years, health technology assessment, proportional shortfall, absolute shortfall, Japan

## Abstract

**Background:**

Since 2019, Japan has implemented health technology assessments (HTAs) for selected drugs and medical devices. In the HTA system, the incremental cost-effectiveness ratio (ICER), calculated using quality-adjusted life-years (QALYs), is employed to guide price adjustments. However, the current system does not incorporate a quantitative assessment of disease severity.

**Objectives:**

This study aimed to evaluate whether severity modifiers based on QALY shortfalls correspond to conditions currently granted special consideration, that is, those eligible for a higher ICER reference value (1.5× the standard), and to explore their implications for Japan’s HTA system.

**Methods:**

We retrospectively analyzed 32 drugs assessed under Japan’s HTA up to March 2025. Absolute shortfall (AS) and proportional shortfall (PS) were calculated using age, sex distribution, and comparator quality-adjusted life expectancy estimates from manufacturer assessments and public assessments. Severity categories were defined as ×1.0 (AS ≤ ×12 or PS ≤0.85), ×1.2 (12 < AS < 18 or 0.85 < PS < 0.95), and ×1.7 (AS ≥18 or PS ≥0.95). The concordance between severity classification and policy-based special consideration was then examined.

**Results:**

Twenty-five matched target populations were identified. Mean AS and PS values did not differ significantly between manufacturer and public assessments, although manufacturers tended to report higher shortfalls. All cancer and pediatric cases were classified as ×1.2 or ×1.7, whereas 1 designated intractable disease was classified as having low severity (×1.0). Chronic and infectious diseases fell into higher severity categories despite not currently being subject to special consideration. Weighted mean severity values were comparable to those used in the UK’s National Institute for Health and Care Excellence benchmarks.

**Discussion:**

The findings revealed both alignment and misalignment between Japan’s current HTA policy and severity classification. While cancer and pediatric diseases were consistent with the existing system, some serious diseases might have been overlooked, and the designated intractable disease might not align with quantitative severity criteria.

**Conclusions:**

QALY shortfalls may serve as a complementary approach to identifying unmet health needs within Japan’s HTA system. To ensure methodological robustness and social acceptance, broader validation, standardized estimation methods, and stakeholder consensus are necessary for effective decision-making.

## BACKGROUND

Advances in medical technology have greatly improved human health, supported life functions, and extended life expectancy. However, healthcare systems face increasing financial pressures, largely due to demographic shifts such as population aging and the growing adoption of advanced medical technologies. Such pressures have raised concerns about the sustainability of healthcare budgets; in response, health technology assessments (HTAs) have been integrated into pricing and reimbursement decision-making processes in many countries. However, HTA systems differ across the world.^1–5^

Since April 2019, Japan has applied an HTA to adjust the prices of drugs and medical devices that meet its selection criteria.^6^ Within this framework, the prices of drugs and medical devices with expected high sales or notably high unit costs are adjusted based on the incremental cost-effectiveness ratio (ICER) derived from cost-effectiveness analyses conducted by manufacturers and public research organizations. Manufacturer assessments are conducted by pharmaceutical or medical device companies to analyze the cost-effectiveness of their own products, following an agreed analytical framework established with an expert committee. Public analyses are conducted by public research organizations or academic groups to review and, when necessary, reanalyze the manufacturers’ submissions.^6^ Quality-adjusted life-years (QALYs) serve as the measure of effectiveness in these cost-effectiveness analyses.^7^ The calculated ICERs are classified into 5 categories, ranging from under 2 million Japanese yen (¥) (US $13 212)/QALY to ¥10 million (US $66 063)/QALY and over. If the ICER is less than ¥5 million (<US $33 031)/QALY, no price adjustment is made. In contrast, if the ICER is at least ¥5 million (≥US $33 031)/QALY, the price is reduced according to a coefficient assigned to each ICER category.^6^ Here, US $1 is converted to ¥151.37, the average exchange rate in 2024.^8^ Meanwhile, drugs and medical devices for cancer, pediatric disease, and designated intractable disease^9^ are currently subject to special consideration, with a higher ICER reference value (1.5× standard) applied.^6^ However, this special consideration is a policy-based regulation rather than an approach grounded in a quantitative assessment of disease severity.

Conversely, Norway, the Netherlands, and the United Kingdom (UK) incorporate quantitative severity, measured by absolute shortfall (AS) and proportional shortfall (PS) of future QALYs lost due to disease, into their HTA systems. Specifically, quantitative severity is used either to adjust the ICER threshold (as in Norway and the Netherlands) or as a multiplicative modifier applied to QALYs, as implemented by the UK’s National Institute for Health and Care Excellence (NICE). In recent years, the QALY shortfall concept, a hybrid framework that integrates the fair innings principle and considerations of severity, has gained increasing attention. Empirical studies, such as that by Stolk et al,^10^ have shown that public preferences often prioritize patients with greater QALY shortfalls, reflecting a societal inclination to favor treatments for individuals experiencing more severe health losses.^11^ By integrating such societal perspectives into their HTA systems, Norway, the Netherlands, and the UK have established institutional mechanisms that promote fairer allocation of medical resources to patient groups with serious conditions.^12–14^

This study assesses the feasibility of incorporating severity modifiers into Japan’s HTA system by examining the relationship between estimated QALY shortfalls, based on the NICE severity modifier framework, and the diseases currently granted special consideration under Japan’s HTA policy.

## METHODS

### Study Population for QALY Shortfall Estimation

We retrospectively estimated QALY shortfalls for drugs evaluated under Japan’s HTA system, applying the severity modifier framework used by NICE. As of March 2025, HTA evaluations had been completed for 32 drugs, with results made publicly available on the Center for Outcomes Research and Economic Evaluation for Health (C2H) website.^15^ These drugs corresponded to 74 target populations. After excluding populations without additional clinical benefits, 49 target populations remained from manufacturer assessments and 48 from public assessments. To accurately calculate AS and PS, we selected only those populations with information on mean age and sex distribution or at least mean age, leaving 26 populations from manufacturer assessments and 26 from public assessments. For populations with missing sex data but available mean age, the sex distribution was imputed as 50% male and 50% female.

**Figure 1.**
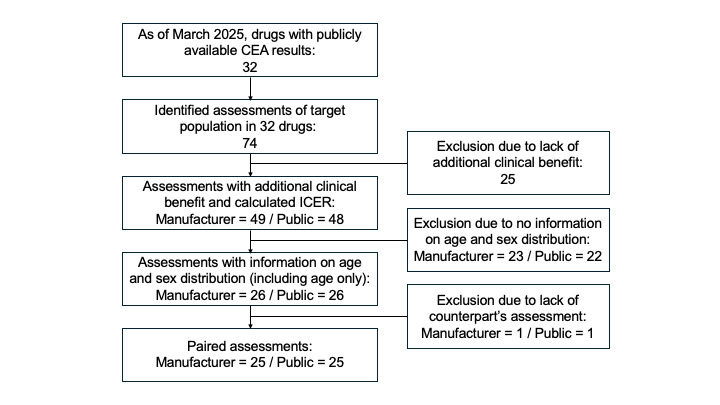
Flow of Selection of Study Populations Abbreviations: CEA, cost-effectiveness analysis; ICER, incremental cost-effectiveness ratio.

**Figure 2.**
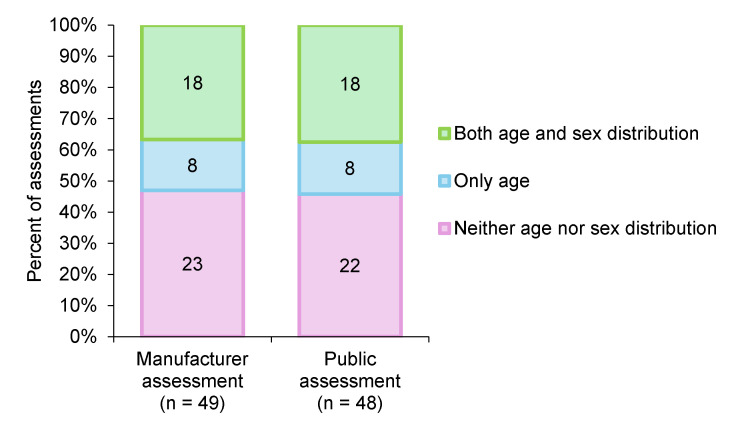
Information Status on Age and Sex Distribution in Manufacturer and Public Assessments Note: Included are 49 target populations from manufacturer assessments and 48 from public assessments, each demonstrating additional clinical benefits.

To determine how input parameters affect QALY shortfall estimates, we compared manufacturer and public assessments, which may differ in the data sources used to define target population characteristics. This comparison allowed us to examine how variations in population attributes, such as age and sex distribution, might influence severity classification outcomes. For this pairwise comparison, a set of 25 matched target populations was established as the study population (**Figure 1**). The information status on age and sex distribution is shown in **Figure 2**.

The mean age and sex distribution of the study population, along with patient quality-adjusted life expectancy (QALE) of the comparator under standard treatment, were systematically extracted from published manufacturer and public assessment documents. To ensure data accuracy, two researchers reviewed the extracted information. For any unclear points, a third researcher was consulted, and consensus was reached through discussion.

### Definition and Calculation of AS and PS

In this study, QALE was defined as the lifetime sum of QALYs. While a QALY represents 1 year of life adjusted for health-related quality of life, QALE reflects the total expected QALYs over an individual’s remaining lifetime. The AS represents the total future health (measured in QALYs) and the PS represents the proportion of future health (expressed in QALYs) that an individual is expected to lose as a result of their condition.^12,16^ AS and PS were calculated as follows:

$$\begin{array}{rl} AS\; & =\;DiscountedQALEoftheGeneralPopulation\\ & \;-DiscountedQALEoftheComparator \end{array}$$

PS=AS/DiscountedQALEoftheGeneralPopulation

**Figure 3.**
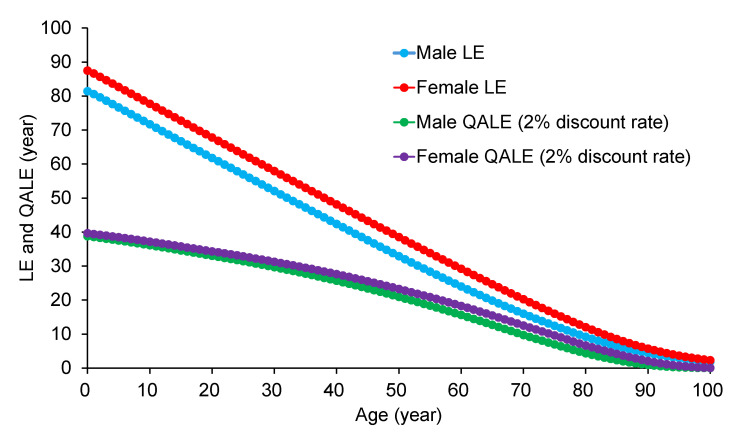
Undiscounted Life Expectancy and Discounted Quality-Adjusted Life Expectancy at Each Age Abbreviations: LE, life expectancy; QALE, quality-adjusted life expectancy. Note: LE was derived from the abridged life tables for Japan, 2019,^18^ and QALE was calculated using Japan’s EQ-5D-5L values^19^ with a 2% discount rate.^20^

The QALE for the general population was estimated using the formula proposed by NICE,^17^ incorporating abridged life tables for 2019^18^ and EQ-5D-5L-based utility values specific to the Japanese population^19^ (**Figure 3** and **Supplementary Table S1**). However, previous studies have not reported utility values for individuals younger than 16 years and older than 90 years. Therefore, we applied utility values for age 16 and 90 to age groups younger than 16 and older than 90, respectively. A 2% annual discount rate was applied in accordance with Japanese HTA guidelines.^20^

Discounted QALE for the general population was calculated^17^ as follows:

$$\sum_{x=i}^{100}Lx\cdot Ux\cdot \frac{1}{(1+r{)}^{x-i}}$$

where *Lx* represents the number of life years lived between age *x* and age *x* + 1, adjusted using a half-cycle correction (ie, the average of survivors at ages *x* and *x* + 1); *Ux* is the age-specific health utility weight derived from EQ-5D-5L population norms; *r* is the annual discount rate (2%); and *i* is the starting age of the target population. Because *Lx* and 𝑈𝑥 differ by sex, calculations were performed separately for males and females, and a weighted mean was computed based on the sex distribution of the target population.

The QALY weighting system for severity, as currently used by NICE, was then applied to the calculated AS and PS scores^21^ as follows:

QALY weight = ×1.0 if AS < 12 or PS < 0.85

QALY weight = ×1.2 if ×12 ≤ AS < 18 or 0.85 ≤ PS < 0.95

QALY weight = ×1.7 if AS ≥18 or PS ≥0.95

Overall severity weights were assigned according to the higher value between AS and PS. Based on the assigned QALY weight, AS, PS, and overall severity were classified into three levels: ×1.0, ×1.2, and ×1.7.^21^

### Statistics

Descriptive statistics were used to summarize attributes of study populations, patient QALYs of the comparator, and estimated AS, PS, and severity weights. Continuous variables are reported as mean ± SD and categorical variables as number and percentage. To identify characteristic differences between manufacturer and public assessments, we compared continuous variables using either the *t* test or Wilcoxon rank-sum test; categorical variables were compared using chi-square or Fisher’s exact tests, as appropriate. All analyses were conducted using Stata SE 17.0 (StataCorp LLC). A *P* value of <.05 was considered statistically significant.

### Cross-Tabulation of Severity Classifications by Disease Category

To assess the alignment between severity classifications based on NICE’s QALY shortfall framework and Japan’s current policy on special consideration, we cross-tabulated severity categories by disease type (eg, cancer, pediatric diseases, and designated intractable diseases).

To further evaluate the alignment between severity classifications based on NICE’s QALY shortfall framework and Japan’s current policy on special consideration, agreement analyses were conducted using Cohen’s kappa. Specifically, 2 × 2 contingency tables were constructed to compare policy-based special consideration (×1.0 vs ×1.5) with QALY shortfall–based severity categories (×1.0 vs ≥×1.2), and the percent agreement and Cohen’s kappa coefficients were calculated. The 95% confidence intervals (CIs) for Cohen’s kappa were estimated using bootstrap resampling (10 000 iterations) because standard functions in Stata 17.0 did not provide reliable confidence interval estimates given the small sample size. Analyses were performed separately for the manufacturer and public assessments, as well as for AS, PS, and overall severity.

## RESULTS

### Comparison of AS and PS Between Manufacturer and Public Assessments

**Table 1** summarizes the characteristics of study populations, estimated AS and PS, and the distribution of severity categories derived from manufacturer and public assessments (n = 25 each).

**Table 1.** Characteristics of Target Population, QALYs of the Comparator, and Distribution of Absolute Shortfall, Proportional Shortfall, and Overall Severity Categories in Manufacturer and Public Assessments

		**Manufacturer Assessment (n = 25)**	**Public Assessment (n = 25)**	***P* Value**
Characteristic				
Age (years), mean (SD)		57.94 (11.21)	60.94 (11.94)	0.36
Female proportion (%), mean (SD)		43 (16)	38 (22)	0.40
QALE of the comparator, mean (SD)		7.539 (6.14)	7.745 (6.54)	0.98
Absolute shortfall				
Mean (SD)		9.863 (5.452)	7.751 (5.002)	0.16
Weight, n (%)	Cat. ×1.0 (AS <12)	15 (60.0)	18 (72.0)	0.19
	Cat. ×1.2 (12 ≤ AS < 18)	7 (28.0)	7(28.0)	
	Cat. ×1.7 (AS ≥18)	3 (12.00)	0 (0.0)	
Proportional shortfall				
Mean (SD)		0.581 (0.294)	0.521 (0.304)	0.48
Weight, n (%)	Cat. ×1.0 (PS <0.85)	18 (72.0)	19 (76.0)	0.92
	Cat. ×1.2 (0.85 ≤ PS < 0.95)	4 (16.0)	3 (12.0)	
	Cat. ×1.7 (PS ≥0.95)	3 (12.0)	3 (12.0)	
Overall				
Mean weight (SD)		1.200 (0.296)	1.116 (0.232)	0.27
Overall weight category, n (%)	Cat. ×1.0	15 (60.0)	18 (72.0)	0.53
	Cat. ×1.2	4 (16.0)	4 (16.0)	
	Cat. ×1.7	6 (24.0)	3 (12.0)	

Abbreviations: Cat., category; QALE, quality-adjusted life expectancy; QALYs, quality-adjusted life-years; SD, standard deviation.

The mean age (± SD) of the study populations was 57.94 ± 11.21 years in manufacturer assessments and 60.94 ± 11.94 years in public assessments, with the latter group being slightly older (*P* = .36). The proportion of female participants was also similar: 43% in manufacturer assessments and 38% in public assessments (*P* = .40). The mean QALYs of the comparator were nearly identical: 7.539 in manufacturer assessments and 7.745 in public assessments (*P* = .98). These results indicate no substantial differences in age, sex distribution, or comparator QALYs between the two study populations.

However, manufacturer assessments tended to yield higher AS and PS scores. The mean AS (± SD) was 9.863 ± 5.452 in manufacturer assessments and 7.751 ± 5.002 in public assessments (*P* = .16). The mean PS (± SD) was 0.581 ± 0.294 and 0.521 ± 0.304, respectively (*P* = .48). A similar trend appeared in the severity classification based on AS: 3 cases were classified in the highest severity category (×1.7) in manufacturer assessments compared with none in public assessments. Based on combined AS and PS results, the overall severity weight placed 10 cases in manufacturer assessments into higher severity categories: 4 in ×1.2 and 6 in ×1.7. In comparison, 7 cases in public assessments fell into higher severity categories, with 4 in ×1.2 and 3 in ×1.7. The weighted mean of the three severity categories was 1.200 in manufacturer assessments and 1.116 in public assessments.

### Association Between Severity Categories and Disease Categories

**Table 2** summarizes the relationship between two types of severity classification: one based on AS and PS using NICE’s methodology and the other based on disease categories designated for special consideration under Japan’s HTA system. This system applies a higher ICER reference value (1.5× the standard) to cancer, pediatric diseases, and designated intractable diseases. Among these, all cancer (n = 6) and pediatric (n = 1) cases were classified as either higher category ×1.2 or the highest severity category ×1.7 in the overall severity classification. In contrast, a designated intractable disease case (n = 1) was classified as ×1.0 in both manufacturer and public assessments. These findings suggest that the diseases granted a higher ICER reference value under the current Japanese HTA system do not always align with the severity defined by NICE’s QALY shortfall framework.

**Table 2.** Distribution of Absolute Shortfall, Proportional Shortfall, and Overall Severity Categories by Disease Category in Manufacturer and Public Assessments

**Disease Category, n (ICER Threshold)**		**Manufacturer Assessment (n = 25)**					**Public Assessment (n = 25)**				
**Total N**	**Disease with Special Consideration**			**Others (n = 17) (×1.0)**	**Total N**	**Disease with Special Consideration**			**Others (n = 17) (×1.0)**		
	**Cancer (n = 6) (×1.5)**	**Pediatric (n = 1) (×1.5)**	**DID (n = 1) (×1.5)**			**Cancer (n = 6) (×1.5)**	**Pediatric (n = 1) (×1.5)**	**DID (n = 1) (×1.5)**			
AS	Cat. ×1.0	15	0	0	1	14	18	0	0	1	17
	Cat. ×1.2	7	5	1	0	1^a^	7	6	1	0	0
	Cat. ×1.7	3	1	0	0	2^b^	0	0	0	0	0
PS	Cat. ×1.0	18	0	1	1	16	19	0	1	1	17
	Cat. ×1.2	4	3	0	0	1^a^	3	3	0	0	0
	Cat. ×1.7	3	3	0	0	0	3	3	0	0	0
Overall	Cat. ×1.0	15	0	0	1	14	18	0	0	1	17
	Cat. ×1.2	4	2	1	0	1^a^	4	3	1	0	0
	Cat. ×1.7	6	4	0	0	2^b^	3	3	0	0	0

Abbreviations: AS, absolute shortfall; Cat., category; DID, designated intractable disease; PS, proportional shortfall.

^a^Heart failure.

^b^Fungal infection.

Analysis of agreement using Cohen’s kappa provided additional insights into this alignment. For manufacturer assessments, agreement was moderate to substantial for AS and overall severity (percent agreement = 84.0%; κ = 0.66; 95% CI, 0.34-0.97 for each) and moderate for PS (86.4%; κ = 0.58; 95% CI, 0.11-1.05), indicating some discrepancies between the Japanese reference value and NICE’s QALY shortfall-based severity classifications. In contrast, public assessments showed stronger alignment, with almost perfect agreement for AS and overall severity (96.0%; κ = 0.90; 95% CI, 0.56-1.08 for each) and substantial agreement for PS (92.0%; κ = 0.80; 95% CI, 0.41–1.03) (**Supplementary Table S2**).

Notably, 3 of 17 cases outside the disease categories currently granted special consideration in Japan exhibited high AS and PS scores in manufacturer assessments. These included 2 infectious diseases and 1 chronic disease, each classified as either ×1.2 or ×1.7 in overall severity.

**Figure 4.**
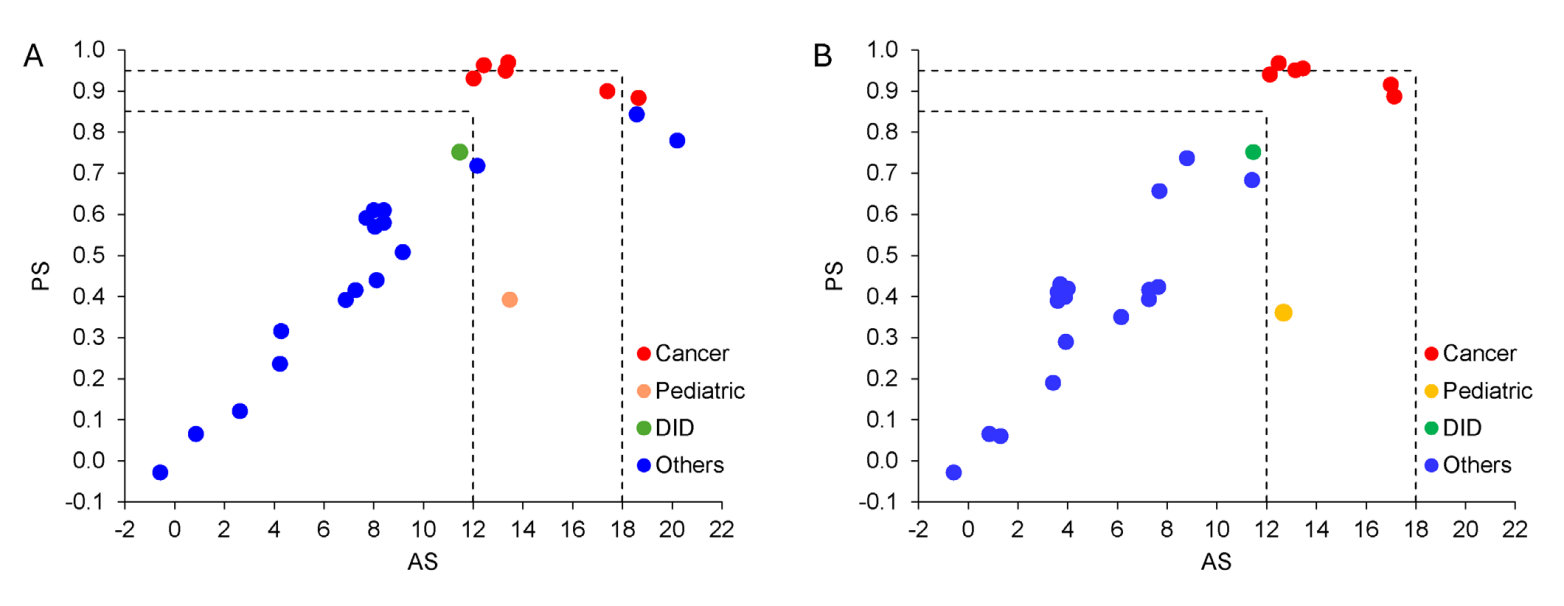
Scatterplots of Estimated AS and PS Values of Target Populations: (**A**) Manufacturer Assessments; (**B**) Public Assessments Abbreviations: AS, absolute shortfall; DID, designated intractable disease; HTA, health technology assessment; NICE, National Institute for Health and Care Excellence; PS, proportional shortfall. Notes: Each dot represents one of the 25 target populations analyzed in each assessment. The dots are color-coded by disease category to visualize the distribution of AS and PS values.Cancer, pediatric disease, and DID are disease categories designated for special consideration currently under the Japanese HTA system.Dashed lines indicate QALY shortfall thresholds used as severity modifiers, as defined by NICE.

The relationship between AS and PS for each target population is further illustrated using scatterplots for both manufacturer and public assessments (**Figure 4**).

## DISCUSSION

This study identified alignment and misalignment between Japan’s current policy on special consideration for certain diseases and severity classifications based on QALY shortfalls estimated from manufacturer and public assessments.

Although the mean AS and PS scores were similar between the two assessments, notable differences appeared in the distribution of severity categories. For AS, 3 cases (12%) in manufacturer assessments were classified in the highest category (×1.7), while no such cases were observed in public assessments. In these discrepant cases, the manufacturer-assessed populations were consistently younger: 41.3 vs 59.9 years, 49.5 vs 68.3 years, and 59.6 vs 68.8 years. Correspondingly, comparator QALYs were also higher in manufacturer assessments: 5.706 vs 5.282, 3.44 vs 3.137, and 4.76 vs 4.01 QALYs. These differences suggest that public assessments, which reflect older populations with shorter life expectancy, show smaller health disparities compared with the general population, resulting in lower AS values. While age is a major factor, the observed discrepancies also stem from differences in data sources and modeling assumptions. Manufacturer assessments typically draw on clinical trial data, which often include younger and healthier participants, whereas public assessments often rely on real-world sources such as the National Database of Health Insurance Claims and Specific Health Checkups of Japan, which consists of all patients, including older individuals and those with multiple comorbidities.^22,23^ Assumptions about long-term survival and utility also vary between models and directly affect QALE estimates. Thus, QALY shortfall calculations are highly sensitive to target population characteristics and modeling choices, with age being especially influential.

Most cancer cases were classified in the higher or highest categories (×1.2 or ×1.7) in AS and PS analyses. Although only one pediatric case was included, it was also classified as ×1.2. These results support the current policy of granting special consideration to certain diseases in Japan’s HTA. By contrast, the designated intractable disease case was classified as low severity (×1.0), suggesting misalignment between policy-based special consideration and quantitative severity assessments based on QALY shortfalls. This implies the possibility of a reduced level of special consideration for diseases requiring ethical and social consideration.

It is noteworthy that 3 cases (ie, 1 chronic disease and 2 infectious diseases) not currently eligible for special consideration under Japan’s system achieved high AS and PS scores in manufacturer assessments and were classified as ×1.2 or higher using NICE’s QALY weighting system. This finding suggests that the current policy may overlook certain diseases that merit prioritization from a QALY shortfall perspective.

The agreement analyses using Cohen’s kappa indicate a high degree of alignment between the Japanese reference value and NICE’s QALY shortfall-based severity classifications. Notably, the public assessments demonstrated a stronger alignment than the manufacturer assessments. Moreover, the weighted mean of the three severity categories derived from Japanese manufacturer assessments (1.200) and public assessments (1.116) was close to the benchmark value (1.119) used in designing NICE’s severity modifier. All three figures were calculated using the same method, applying severity weights (×1.0, ×1.2, and ×1.7) to the distribution of disease severity. In NICE’s case, this benchmark was based on a substantially larger dataset containing 364 HTA evaluations conducted between April 2011 and November 2019, distributed as 223 (61.3%) in severe weight ×1.0, 111 (30.5%) in ×1.2, and 30 (8.2%) in ×1.7.^24^ However, the distribution of disease severity in Japan differs from that in the UK, and the numerical similarity between the two countries may therefore be coincidental, reflecting differences in HTA objectives and the types of products evaluated. Importantly, NICE identified AS and PS and the corresponding QALY weights through a retrospective analysis of 364 HTA decisions, which served as the primary evidence base for determining thresholds and weightings for the new modifier.^24^ Japan should develop its own benchmark values that reflect the specific goals of its HTA system, supported by a sufficient number of validated cases to ensure methodological robustness.

When considering the incorporation of severity modifiers based on QALY shortfalls into Japan’s HTA framework, a carefully designed stepwise approach is essential to address several key issues. First, it is necessary to build ethical and social consensus on the justification for priority setting, particularly in balancing the needs of older vs younger populations and patients with severe vs mild conditions. Second, standardized methods for estimating QALY shortfalls must be developed. A particular challenge is the lack of Japan-specific utility values for target populations and comparators under standard treatment,^25^ which limits the accuracy of QALY shortfall estimates for the Japanese population. Third, to incorporate AS and PS results into adjustment of ICER reference value, it is necessary to specifically design and validate appropriate reference value and their corresponding adjustment coefficients. This process should be supported by further validation across a broader and sufficiently large set of HTA cases to ensure robustness and generalizability.

Several countries have explored candidate QALY shortfalls as a means for quantifying disease severity. Ultimately, Norway, the Netherlands, and the UK (NICE) adopted AS, PS, and a combination of both, respectively. In these countries, the introduction of QALY shortfall has been carefully considered not only as a tool for improving healthcare efficiency but also as a mechanism for applying ethical principles, such as “Fair Innings” and the “Rule of Rescue,” to decisions about healthcare resource allocation.^13,14,17,26^ For example, during system development, NICE solicited public comments from a broad range of stakeholders, including patient organizations, the pharmaceutical industry, and the general public, and repeatedly deliberated on the ethical, clinical, and policy implications of the proposed changes.^27^ Such a process that prioritizes transparency and consensus building is critical for strengthening societal acceptance of the institutional framework.

This study has several limitations. First, approximately half of the reported HTA cases were excluded, as only those with available information on age and sex distribution were analyzed. As a result, the number of matched manufacturer and public assessments was limited to 25. Among these, only one case involved a pediatric disease and, one involved a designated intractable disease, which constrains the interpretation of results for these disease categories. Thus, the small sample size and limited disease representation may constrain the external validity of our findings, as they may not fully capture the characteristics of HTA cases in Japan. Future research should validate these findings using a larger and more diverse dataset to enhance generalizability. Second, the QALE of the general population was estimated using the 2019 abridged life table^18^ and EQ-5D-5L-based utility values^19^ specific to the Japanese population. These values were uniformly applied across all cases. However, QALE estimates vary depending on the choice of life table, utility values, discount rate, and calculation method, and AS and PS values may differ under alternative assumptions. NICE has investigated how methodological and data source differences influence outcomes and emphasized the importance of standardizing calculation methods.^21^ Furthermore, to ensure accuracy and transparency in QALY shortfall estimation, future HTAs should extract age and sex data for target populations using a standardized reporting format.^17^ Third, sensitivity analyses were not conducted to test the robustness of the results against variations in key assumptions and input parameters. Such analyses could offer additional insights. However, this study primarily aimed to examine the conceptual alignment between Japan’s qualitative severity considerations and NICE’s quantitative severity categories, rather than to validate parameter-level robustness. Future research should incorporate sensitivity analyses when developing Japan-specific shortfall estimation methods and severity coefficients. Finally, because public assessments follow manufacturer submissions, any differences between them may have been influenced by this sequence. Nonetheless, an independent appraisal is conducted to provide a comprehensive evaluation using both assessments.

## CONCLUSION

This study suggests that QALY shortfalls provide a complementary approach to identifying unmet health needs that are overlooked under the current system. However, such a shift would not be uniformly advantageous: while it could extend benefits to previously neglected populations, it may simultaneously reduce advantages for others who have been recognized under the existing framework. Therefore, integrating QALY shortfalls into policy will require further validation based on a broader range of HTA cases. A transparent, stepwise process of consensus building among stakeholders is essential for ensuring methodological robustness and social acceptability.

### Disclosures

M.E. and A.Y. are full-time employees of Pfizer Japan Inc. S.I. has no conflicts of interest to declare.

## Supplementary Material

Online Supplementary Material

## Data Availability

The datasets generated during and/or analyzed during the current study are available from the corresponding author on reasonable request.
