# Supplementary material for: Quantifying Disease Severity in Health Technology Assessment in Japan: A Retrospective Analysis Using Quality-Adjusted Life-Year Shortfalls
Source: J Health Econ Outcomes Res. 2025 Dec 2;12(2):229–36. doi: 10.36469/001c.147469 (PMC12677002; doi:10.36469/001c.147469)
Supplement: Online Supplementary Material [file jheor_2025_12_2_147469_318079.pdf]

Journal of Health Economics and Outcomes Research

Online Supplementary Material

Quantifying Disease Severity in Health Technology Assessment in Japan: A Retrospective Analysis Using Quality-Adjusted Life-Year Shortfalls. *JHEOR*. 2025;12(2):229-236. doi:10.36469/jheor.2025.247469

Table S1: Undiscounted Life Expectancy and Discounted Quality-Adjusted Life Expectancy at Each Age

Table S2: Agreement Between Policy-Based Special Consideration and QALY Shortfall–Based Classification

This supplementary material has been provided by the authors to give readers additional information about their work.

This is an open-access article distributed under the terms of the Creative Commons Attribution 4.0 International License (CCBY-4.0). View this license's legal deed at http://creativecommons.org/licenses/by/4.0 and legal code at http://creativecommons.org/licenses/by/4.0/legalcode for more information.

Table S1. Undiscounted Life Expectancy and Discounted Quality-Adjusted Life Expectancy at Each Age

Age, y	Male LE	Male QALE	Female LE	Female QALE
0	81.41	38.769	87.450	39.621
1	80.57	38.525	86.600	39.394
2	79.59	38.278	85.630	39.164
3	78.61	38.026	84.640	38.930
4	77.62	37.769	83.650	38.691
5	76.63	37.507	82.660	38.447
6	75.63	37.240	81.670	38.198
7	74.64	36.968	80.680	37.945
8	73.65	36.690	79.680	37.687
9	72.65	36.407	78.690	37.423
10	71.66	36.118	77.690	37.155
11	70.66	35.824	76.690	36.881
12	69.67	35.523	75.700	36.601
13	68.67	35.217	74.700	36.316
14	67.68	34.905	73.710	36.026
15	66.69	34.587	72.720	35.729
16	65.7	34.262	71.730	35.427
17	64.71	33.953	70.730	35.153
18	63.73	33.639	69.740	34.873
19	62.75	33.318	68.760	34.588
20	61.77	32.992	67.770	34.297
21	60.8	32.687	66.780	34.015
22	59.83	32.376	65.800	33.727
23	58.85	32.059	64.810	33.434
24	57.88	31.736	63.820	33.135
25	56.91	31.408	62.840	32.830
26	55.93	31.073	61.850	32.520
27	54.96	30.732	60.860	32.203
28	53.98	30.384	59.880	31.881
29	53.01	30.030	58.890	31.552
30	52.03	29.670	57.910	31.217
31	51.06	29.301	56.920	30.884
32	50.09	28.925	55.940	30.546
33	49.12	28.542	54.960	30.200
34	48.15	28.151	53.980	29.848
35	47.18	27.754	53.000	29.490
36	46.21	27.349	52.020	29.124
37	45.24	26.937	51.040	28.752
38	44.28	26.517	50.060	28.372
39	43.31	26.090	49.080	27.986
40	42.35	25.655	48.110	27.592
41	41.39	25.218	47.140	27.190
42	40.43	24.773	46.170	26.780
43	39.47	24.321	45.200	26.363
44	38.52	23.860	44.230	25.938
45	37.57	23.392	43.260	25.505

Table S1. Undiscounted Life Expectancy and Discounted Quality-Adjusted Life Expectancy at Each Age

Age, y	Male LE	Male QALE	Female LE	Female QALE
46	36.62	22.915	42.300	25.065
47	35.68	22.430	41.340	24.616
48	34.74	21.937	40.390	24.160
49	33.81	21.437	39.440	23.696
50	32.89	20.928	38.490	23.223
51	31.97	20.426	37.540	22.762
52	31.05	19.918	36.600	22.294
53	30.14	19.401	35.660	21.818
54	29.24	18.877	34.730	21.333
55	28.34	18.346	33.790	20.841
56	27.45	17.808	32.860	20.341
57	26.57	17.262	31.930	19.832
58	25.7	16.710	31.000	19.316
59	24.83	16.151	30.080	18.791
60	23.97	15.586	29.170	18.259
61	23.13	15.017	28.250	17.716
62	22.29	14.441	27.340	17.165
63	21.46	13.861	26.430	16.607
64	20.64	13.276	25.530	16.040
65	19.83	12.688	24.630	15.465
66	19.03	12.095	23.730	14.883
67	18.24	11.501	22.840	14.293
68	17.47	10.904	21.960	13.696
69	16.71	10.306	21.080	13.091
70	15.96	9.709	20.210	12.480
71	15.23	9.147	19.350	11.910
72	14.51	8.587	18.490	11.334
73	13.79	8.031	17.640	10.753
74	13.09	7.478	16.800	10.168
75	12.41	6.932	15.970	9.579
76	11.73	6.392	15.150	8.987
77	11.08	5.860	14.340	8.393
78	10.43	5.338	13.550	7.797
79	9.8	4.828	12.770	7.201
80	9.18	4.330	12.010	6.607
81	8.59	3.891	11.260	6.100
82	8.02	3.469	10.540	5.598
83	7.48	3.065	9.840	5.103
84	6.95	2.681	9.160	4.617
85	6.46	2.320	8.510	4.143
86	5.99	1.984	7.890	3.682
87	5.55	1.674	7.300	3.239
88	5.14	1.393	6.730	2.816
89	4.76	1.141	6.210	2.417
90	4.41	0.919	5.710	2.044
91	4.08	0.726	5.240	1.700

Table S1. Undiscounted Life Expectancy and Discounted Quality-Adjusted Life Expectancy at Each Age

Age, y	Male LE	Male QALE	Female LE	Female QALE
92	3.76	0.562	4.800	1.387
93	3.47	0.424	4.390	1.107
94	3.19	0.312	4.000	0.860
95	2.94	0.222	3.640	0.648
96	2.7	0.151	3.310	0.469
97	2.47	0.098	3.020	0.322
98	2.26	0.059	2.750	0.205
99	2.07	0.031	2.510	0.115
100	1.89	0.012	2.290	0.048

Abbreviations: LE, life expectancy; QALE, quality-adjusted life expectancy.
LE was based on the abridged life tables for Japan, 2019,[18] and QALE was calculated using Japan's EQ-5D-5L values[19] and a 2% discount rate.[20]

Table S2. Agreement Between Policy-Based Special Consideration and QALY Shortfall–Based Classification

		Other Diseases, N	Disease with Special Consideration, N	Total, N	Agreement	Cohen's Kappa Value (95% CI,[a] *P* Value)
(ICER Threshold)		(×1.0)	(×1.5)			
Manufacturer assessment						
AS	Cat. ×1.0, N	14	1	15	84.0%	0.66 (0.34-0.97, $P < .001$)
	Cat. ×1.2 or ×1.7, N	3	7	10		
	Total, N	17	8	25		
PS	Cat. ×1.0, N	16	2	18	86.4%	0.58 (0.11-1.05, $P < .01$)
	Cat. ×1.2 or ×1.7, N	1	6	7		
	Total, N	17	8	25		
Overall	Cat. ×1.0, N	14	1	15	84.0%	0.66 (0.34-0.97, $P < .001$)
	Cat. ×1.2 or ×1.7, N	3	7	10		
	Total, N	17	8	25		
Public assessment						
AS	Cat. ×1.0, N	17	1	18	96.0%	0.90 (0.56-1.08, $P < .001$)
	Cat. ×1.2 or ×1.7, N	0	7	7		
	Total, N	17	8	25		
PS	Cat. ×1.0, N	17	2	19	92.0%	0.80 (0.41-1.03, $P < .001$)
	Cat. ×1.2 or ×1.7, N	0	6	6		
	Total, N	17	8	25		
Overall	Cat. ×1.0, N	17	1	18	96.0%	0.90 (0.56-1.07, $P < .001$)
	Cat. ×1.2 or ×1.7, N	0	7	7		
	Total, N	17	8	25		

Abbreviations: AS, absolute shortfall; Cat., category; CI, confidence interval; PS, proportional shortfall; QALY, quality-adjusted life-year..
[a]95% CI estimated using bootstrap resampling (10 000 iterations).
